# Collagen IV of basement membranes: I. Origin and diversification of *COL4* genes enabling metazoan multicellularity, evolution, and adaptation

**DOI:** 10.1016/j.jbc.2025.108496

**Published:** 2025-04-11

**Authors:** Patrick S. Page-McCaw, Elena N. Pokidysheva, Carl E. Darris, Sergei Chetyrkin, Aaron L. Fidler, Julianna Gallup, Aspirnaut Team, Julianna Gallup, Jillian Balser, Cullen Curbow, Ricard Harris, Cody Stothers, Keith Wade, Prayag Murawala, Julie K. Hudson, Sergei P. Boudko, Billy G. Hudson

**Affiliations:** 1 Department of Cell and Developmental Biology, Vanderbilt University, Nashville, Tennessee, USA; 2 Division of Nephrology and Hypertension, Department of Medicine, Vanderbilt University Medical Center, Nashville, Tennessee, USA; 3 Aspirnaut Program, Vanderbilt University Medical Center, Nashville, Tennessee, USA; 4 Center for Matrix Biology, Vanderbilt University Medical Center, Nashville, Tennessee, USA; 5 Mass Spectrometry Research Center, Vanderbilt University, Nashville, Tennessee, USA; 6 Mount Desert Island Biological Laboratory, Bar Harbor, Maine, USA; 7 Department of Nephrology and Hypertension, Hannover Medical School, Hannover, Germany; 8 Department of Medical Education and Administration, Vanderbilt University Medical Center, Nashville, Tennessee, USA; 9 Department of Biochemistry, Vanderbilt University, Nashville, Tennessee, USA; 10 Vanderbilt-Ingram Cancer Center, Vanderbilt University, Nashville, Tennessee, USA; 11 Vanderbilt Institute of Chemical Biology, Vanderbilt University, Nashville, Tennessee, USA; 12 Department of Biological Sciences, Vanderbilt University, Nashville, Tennessee, USA; 13 Department of Pathology, Microbiology, and Immunology, Vanderbilt University Medical Center, Nashville, Tennessee, USA; 14 Vanderbilt University Medical Center, Vanderbilt University, Nashville, Tennessee, USA

**Keywords:** collagen IV, basement membrane, metazoan evolution, glomerular filtration barrier, Alport syndrome

## Abstract

Collagen IV (Col-IV) is a major component of basement membranes, a specialized form of extracellular matrix that enabled the assembly of multicellular epithelial tissues. In mammals, Col-IV assembles from a family of six α-chains (α1–α6), forming three supramolecular scaffolds: Col-IV**^α121^**, Col-IV**^α345^**, and Col-IV**^α121–α556^**. The α-chains are encoded by six genes (*COL4A*1–6) that occur in pairs in a head-to-head arrangement. In Alport syndrome, variants in *COL4A*3, 4, or 5 genes, encoding Col-IV**^α345^** scaffold in glomerular basement membrane (GBM), the kidney ultrafilter, cause progressive renal failure in millions of people worldwide. The molecular mechanisms of how variants cause dysfunction remain obscure. Here, we gained insights into Col-IV**^α345^** function by determining its evolutionary lineage, as revealed from phylogenetic analyses and tissue expression of *COL4* gene pairs. We found that the *COL4A*⟨1|2⟩ gene pair emerged in basal Ctenophores and Cnidaria phyla and is highly conserved across metazoans. The *COL4A*⟨1|2⟩ duplicated and arose as the progenitor to the *COL4A*⟨3|4⟩ gene pair in cyclostomes, coinciding with emergence of kidney GBM, and expressed and conserved in jawed vertebrates, except for amphibians, and a second duplication as the progenitor to the *COL4A*⟨5|6⟩ gene pair and conserved in jawed vertebrates. These findings revealed that Col-IV**^α121^** is the progenitor scaffold, expressed ubiquitously in metazoan basement membranes, and which evolved into vertebrate Col-IV**^α345^** and expressed in GBM. The Col-IV**^α345^** scaffold, in comparison, has an increased number of cysteine residues, varying in number with osmolarity of the environment. Cysteines mediate disulfide crosslinks between protomers, an adaptation enabling a compact GBM that withstands the high hydrostatic pressure associated with glomerular ultrafiltration.

Collagen IV (Col-IV) is a principal component of basement membranes, a specialized form of extracellular matrix that enabled the genesis and evolution of multicellular epithelial tissues (1, 2). In pioneering studies of the glomerular basement membrane (GBM) of canine and bovine kidneys, Col-IV was identified as a novel collagen and shown to be structurally altered in diabetic kidney disease (DKD) (3, 4, 5, 6, 7, 8, 9). It was first characterized as a supramolecular network of triple helical protomers composed of α1 and α2 chains (10, 11). In subsequent studies of the GBM in Goodpasture’s disease (GP) and Alport syndrome (AS; Fig. 1*A*), four additional chains were discovered, α3–α6 (Fig. 1*B*) (12, 13, 14, 15, 16, 17, 18, 19, 20). The chains are encoded by six genes (*COL4A1*–*COL4A6*), which are located in gene pairs (*COL4A*⟨1|2⟩, *COL4A*⟨3/4⟩, and *COL4A*⟨5/6⟩), in a head-to-head arrangement on three different chromosomes (Fig. 1*D*) (21, 22). In mammals, the α-chains coassemble into heterotrimers, called protomers, of three distinct molecular compositions: α121, α345, and α565, which in turn assemble into three distinct supramolecular scaffolds, referred to as Col-IV**^121^**, Col-IV**^α345^**, and Col-IV**^α121–α556^** (23, 24).

**Figure 1:**
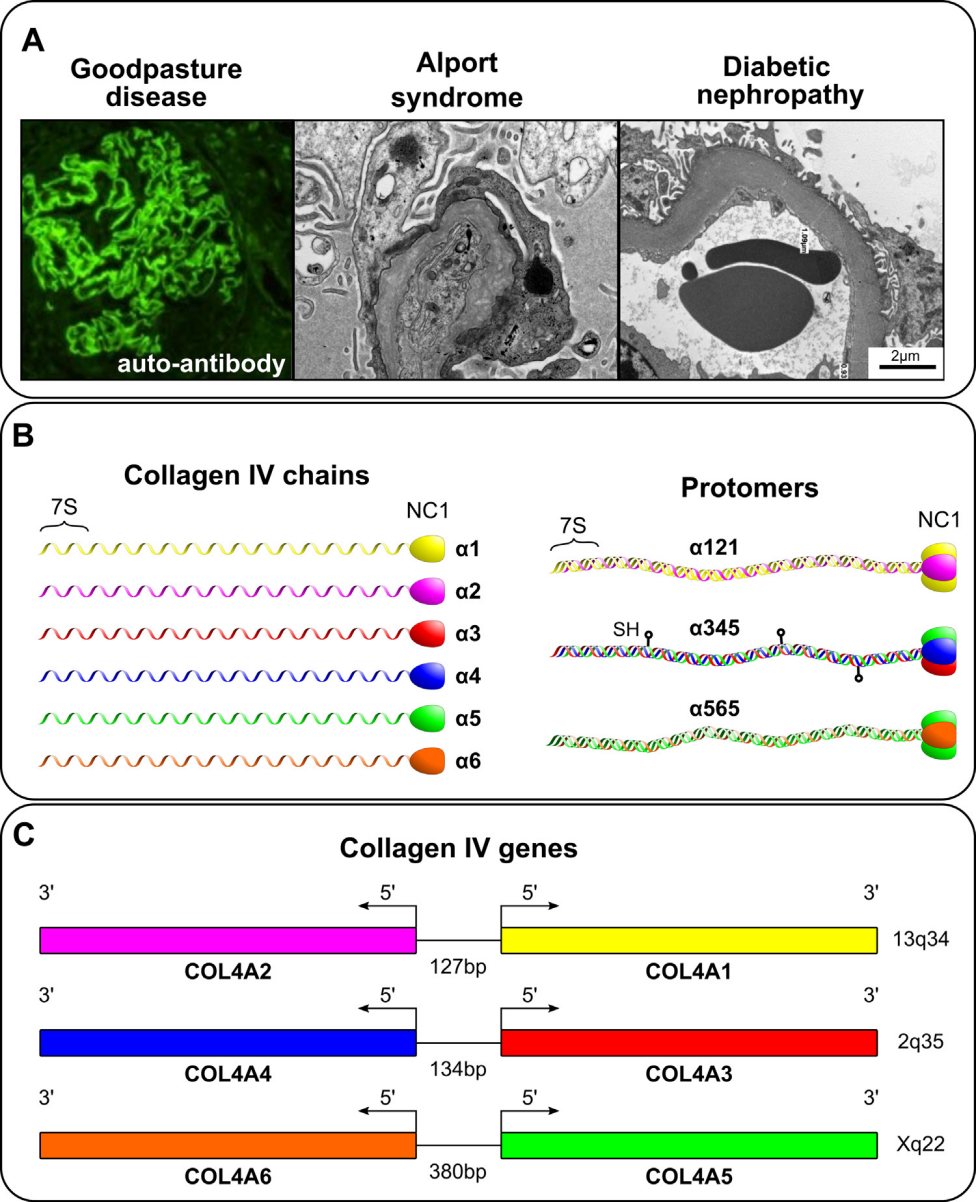
**Collagen IV (Col-IV)-related kidney diseases, protomer, and gene organizations in mammals.***A*, represents characteristic images of GBM abnormalities in three major diseases: Goodpasture's disease, an autoimmune disorder, which is diagnosed based on the linear immunofluorescent GBM staining; Alport syndrome, a genetic disorder, where GBM is split with a characteristic basket weaving; diabetic nephropathy, a common complication of diabetes, where GBM is dramatically thickened. In each of these pathologies, Col-IV is affected. *B*, six protein chains of Col-IV are produced in mammals. These six chains assemble into trimeric complexes referred to as protomers. The N- and C-terminal 7S and noncollagenous (NC1) domains mediate end-to-end homomeric interactions between protomers generating the different Col-IV scaffolds. Both 7S–7S interprotomer tetramers and NC1–NC1 interprotomer dimers are covalently crosslinked. The Col-IV**^α345^** protomer encodes multiple Cys residues, most of which (15) are found within the triple helical domain of alpha 4 chain, which likely form lateral crosslinks between different Col-IV**^α345^** collagen domains. The evolution of the cysteine residues is discussed later in this article. *C*, the head-to-head arrangement of the three COL4A gene pairs found in mammals is shown. Their human chromosomal location is indicated, and the distances between their transcriptional start sites is shown. GBM, glomerular basement membrane.

The Col-IV**^α121^** scaffold is a ubiquitous component of mammalian basement membranes. For example, in the nephron, this scaffold occurs in the GBM, mesangial matrix, and basement membranes of Bowman’s capsule, tubules, and capillaries (Fig. 2) (25, 26). The scaffold confers tensile strength and acts as a tether for diverse macromolecules, including laminin, nidogen, proteoglycans, and growth factors, forming the supramolecular complexes that interact with cell surface receptors (2, 27, 28, 29). Disrupting this scaffold causes basement membrane destabilization and tissue dysfunction in early mouse development (30). Genetic defects of the Col-IV**^α121^** scaffold cause Gould syndrome, porencephaly, and HANAC syndrome in humans (31, 32, 33).

**Figure 2:**
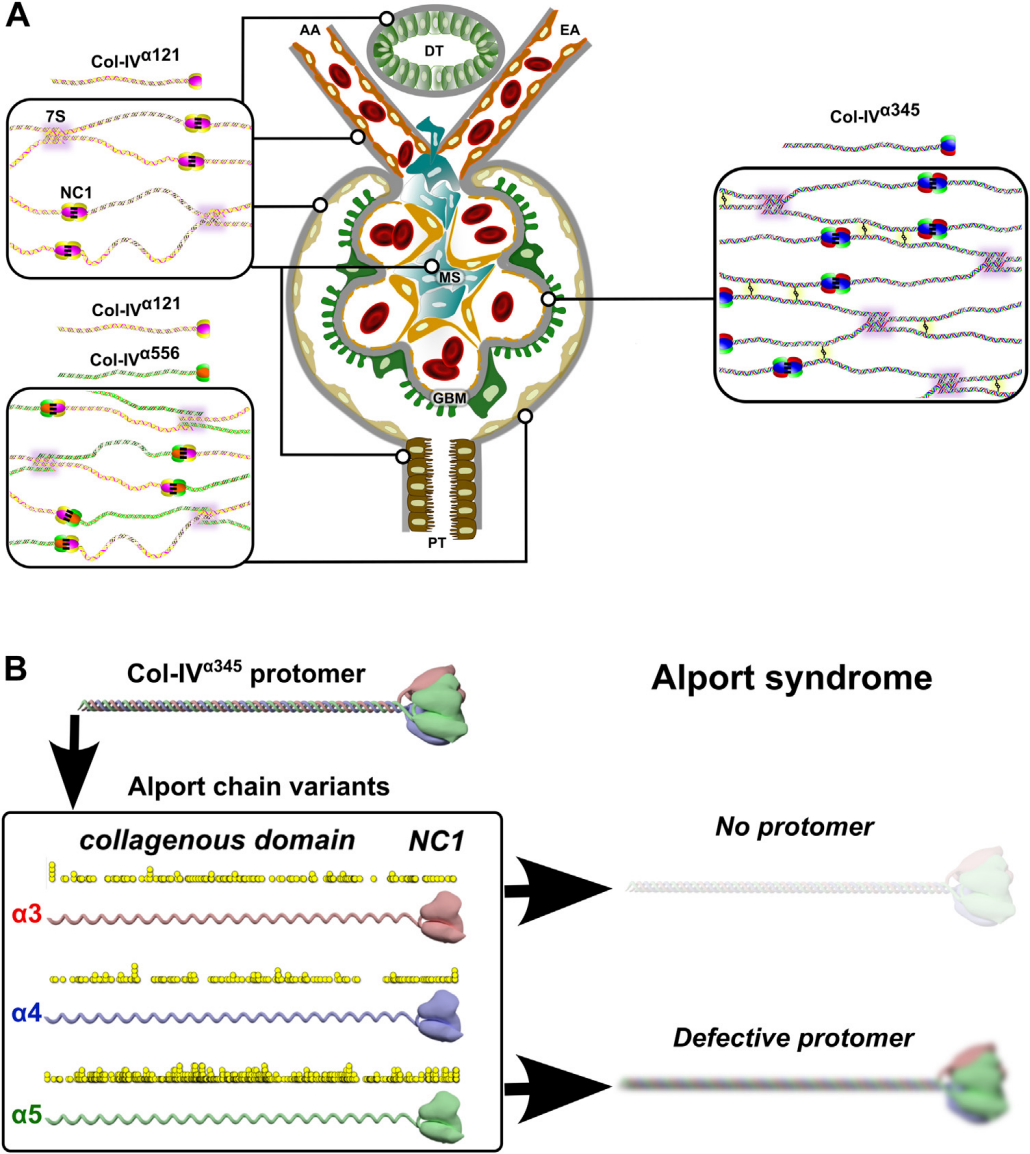
**Collagen IV (Col-IV) scaffolds of the mammalian kidney glomerulus.***A*, three distinct supramolecular Col-IV scaffolds, noted as Col-IV**^α121^**, Col-IV**^α345^**, and Col-IV**^α121–α556^**, comprise the mammalian kidney glomerulus. Scaffolds are assembled from three different triple-helical protomers having three molecular compositions of a-chains: α121, α345, and α565. Protomers are characterized by a 7S domain at the N terminus, a long collagenous domain of Gly-Xaa-Yaa (GXY) repeats of ∼1400 residues with interruptions in the GXY repeats, followed by a noncollagenous (NC1) domain at the C terminus of approximately ∼230 residues. Scaffold assembly steps include dimerization of NC1 trimers and tetramerization of 7S trimers (10). In addition to these interactions, Col-IV**^α121^** scaffold also possesses lateral associations with formation of supercoils (11). The Col-IV**^α121^** scaffold is a component of the basement membranes surrounding tubules, arterioles, and Bowman’s capsule. It is also found within the mesangial space. Uniquely, Bowman’s capsule contains the heteroscaffold of Col-IV**^α121–α556^**. The glomerular corpuscle consists of looped capillaries lined with fenestrated endothelial cells, the glomerular basement membrane (GBM), and podocytes. The Col-IV**^α345^** scaffold is the major component of GBM; it is reinforced by disulfide bonds, which form lateral crosslinks between protomers. *B*, mutations in any of the *COL4A3*, *A4*, or A5 genes that encode the Col-IV**^α345^** scaffold cause Alport syndrome. Known disease variants are mapped onto the predicted protein structures as *yellow circles*. Disease variants cause either the assembly of defective protomers or their complete absence of assembled protomers. AA, afferent arteriole; DT, distal tubule; EA, efferent arteriole; MS, mesangial space; PT, proximal tubule.

Conversely, the Col-IV**^α345^** scaffold, encoded by *COL4A*⟨3/4⟩ and *COL4A*⟨5/6⟩ gene pairs, has a restricted tissue distribution in the lens capsule, retina, inner ear, testis, and kidney (34). In the nephron, Col-IV**^α345^** is the major component of the GBM, a critical morphological feature that functions as an ultrafilter of proteins (Fig. 2) (25, 26, 34). In GP, affecting thousands of people worldwide, autoantibodies target neoepitopes in the Col-IV**^α345^** scaffold causing rapidly progressive renal failure (19, 35, 36, 37, 38, 39, 40, 41). In DKD, affecting millions of people, this scaffold is also involved in the thickening of the GBM morphology that is associated with progressive kidney failure. In AS, affecting millions of people, currently over 5000 genetic variants occur in the *COL4A3*, *COL4A4*, and *COL4A5* genes (according to the ClinVar database (42)) (15, 43, 44, 45, 46, 47). Variants cause either loss of scaffold from the GBM or assembly of a defective scaffold, causing proteinuria and progression to kidney failure (26, 48). A knowledge of how the Col-IV**^α345^** scaffold functions at the molecular level is critical to understanding the pathogenesis of GP, DKD, and AS, thus providing a framework for the development of therapies.

Here, we sought to gain insights into Col-IV**^α345^** function by determining its evolutionary lineage with the Col-IV**^α121^** scaffold, as evinced from phylogenetic analyses, gene synteny, and tissue expression of *COL4* gene pairs. The recent availability of a plethora of high-quality genome assemblies provided an approach to trace the evolutionary emergence of *COL4* gene pairs. The findings revealed that Col-IV**^α121^** scaffold is the progenitor, expressed ubiquitously in basement membranes in all animals, and that evolved into vertebrate Col-IV**^α345^** scaffold with expression mainly in the GBM. The Col-IV**^α345^** scaffold differs from Col-IV**^α121^** by an increased number of lateral disulfide crosslinks, indicating an evolutionary adaptation that enabled the assembly of a compact GBM that withstands the high hydrostatic pressure associated with glomerular ultrafiltration.

## Results

### Phylogenetic analysis of the evolutionary lineage of *COL4* gene pairs

We sought to determine the evolutionary lineage of the *COL4* gene pairs, using the approach of comparative genomics and syntenic relationships. DNA sequence is conserved within orthologous and paralogous genes, which allows evolutionary relationships between genes to be determined. Analogously, gene content of chromosomes, called synteny, is also conserved even across large evolutionary distances (49, 50). Moreover, gene order and orientation, collinearity, is often conserved in microsyntenic blocks. Microsyntenic conservation can provide information about the origin of gene families following speciation and chromosomal duplication. Importantly, recent advances in sequencing technology have facilitated the analysis of gene order and locus structure in diverse species (51). These advances have been accompanied by improvements and standardization in the presentation of genome structure that facilitates an informatics approach to decipher the arrangement and genetic lineage of the *COL4* gene family.

**Figure 3:**
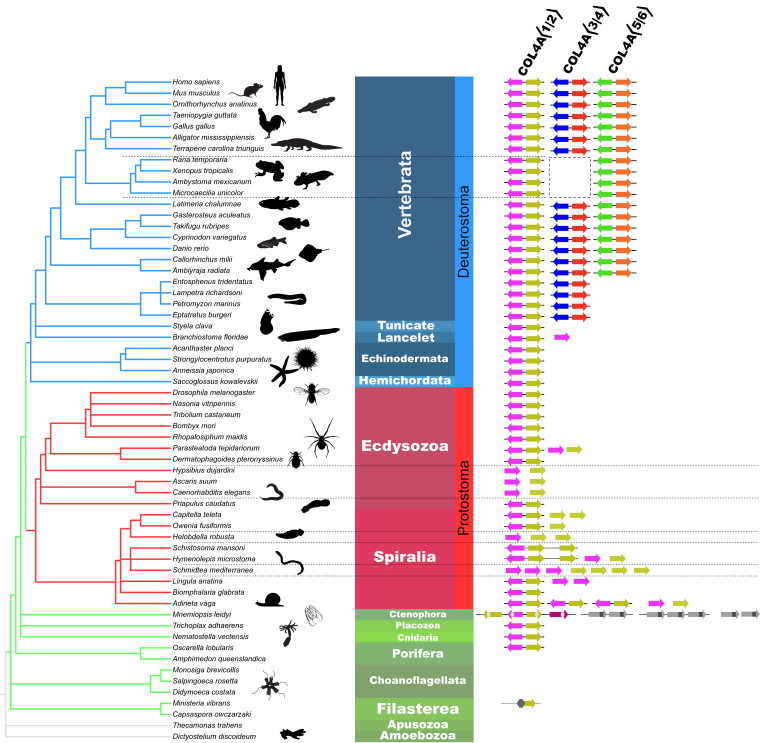
**An evolutionary lineage of COL4A genes that gave rise to the appearance of the COL4A⟨3|4⟩ and COL4A⟨5|6⟩ gene pairs by gene duplication in vertebrate animals.** The eukaryotic cladogram with selected species showing evolution of the COL4A gene pair family mapped onto the National Center for Biotechnology Information Taxonomy (79). The cladogram is rendered using PhyloT v2 and iTOL v6 (76). Animal silhouettes in this and subsequent figures are downloaded from PhyloPic.

Prior studies (21, 52, 53) established that the six mammalian *COL4* genes are arranged in head-to-head pairs, noted as *COL4A*⟨1|2⟩, *COL4A*⟨3|4⟩, and *COL4A*⟨5|6⟩ gene pairs (Fig. 1*C*). We searched diverse genomes across metazoa for each of these gene pairs. First, we found the *COL4A*⟨1|2⟩ gene pair to be conserved across metazoans, with only a few exceptions in the Protosome lineage (Fig. 3). In the nematode clade, for example, the *COL4A1* and *COL4A2* genes (in *Caenorhabditis elegans*, the genes emb-9 and let-2) occur as single and unlinked loci. In other examples, the planarians have multiple copies of single and unlinked *COL4* genes, and *Owenia fusiformis* has both the *COL4A*⟨1|2⟩ gene pair and a *COL4* single gene. Other Protostomes, for example, *Schistosoma mansoni*, have gene arrangements similar to that in Ctenophores in which the *COL4* loci include a total of four *COL4* genes with each set arranged head to tail and a *COL4A*⟨1|2⟩ gene pair positioned adjacent to a single *COL4* gene. Second, we found that the *COL4A*⟨3|4⟩ gene pair emerged in hagfish and lamprey lineages and was conserved in all vertebrates, except for amphibians. Third, the *COL4A*⟨5|6⟩ gene pair emerged in cartilaginous fish and is conserved in all vertebrates (Fig. 3). Collectively, our findings indicate that the *COL4A*⟨1|2⟩ gene pair is the progenitor of vertebrate *COL4A*⟨3|4⟩ and that either *COL4A*⟨1|2⟩ or *COL4A*⟨3|4⟩ is the progenitor of the *COL4A*⟨5|6⟩ gene pair.

We propose a plausible path of how diverse *COL4A1* and *A2* gene arrangements arose in Protostomes (Fig. S1). The gene duplication events, resulting in multiple copies of the *COL4* gene pairs as found in *Adineta vega*, may also lead to loss of one of the *COL4*⟨1|2⟩ pairs either through incomplete gene duplications or accumulation of missense or nonsense alleles in redundant *COL4* genes. This may result in the presence of the original *COL4A*⟨1|2⟩ gene pair and one or more *COL4* single copy genes, such as observed in *Capitella teleta*. The *COL4A*⟨1|2⟩ gene pair could, finally, be lost in some species resulting in two, or more, single copies of the *COL4* gene as observed in *C*. *elegans*. While other evolutionary paths may be possible, this gene duplication hypothesis is consistent with an ancient and conserved *COL4A*⟨1|2⟩ gene pair, and its ancestral role in the emergence of vertebrate *COL4A*⟨3|4⟩ and the *COL4A*⟨5|6⟩ gene pairs.

**Figure 4:**
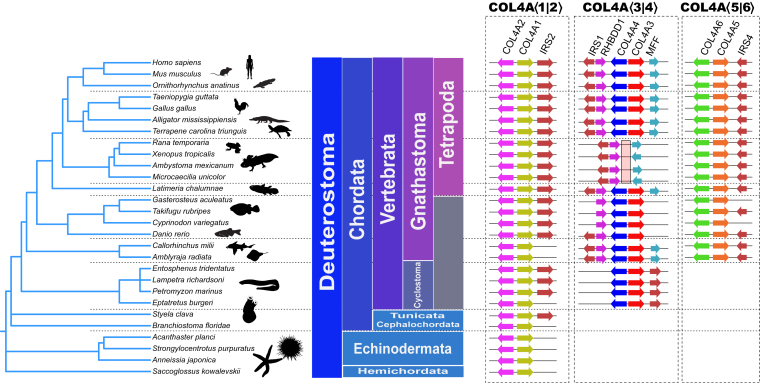
**Duplications of the COL4A⟨1|2⟩ gene pair in deuterostomes and vertebrates.** Basal vertebrates, such as starfish, acorn worms, lancelets, and tunicates, have a single COL4A⟨1|2⟩ gene pair as found in basal metazoans. In most deuterostomes, the COL4A⟨1|2⟩ gene pair (*yellow* and *magenta*) is found tightly linked to an IRS2 ortholog (*light blue*). Notably, the tunicate *Styela clava* has an IRS ortholog located approximately 14 MBp from the COL4A⟨1|2⟩ gene pair. COL4A⟨3|4⟩ gene pair (*red* and *blue*) first appears in cyclostomes and is found in all vertebrate clades except amphibians, often near the IRS1 (*light blue*) and RHBDD1 (*gray*) orthologs. The COL4A⟨5|6⟩ (*orange* and *green*) gene pair is first observed in bony fishes including basal chondrichthyes and is tightly linked to the IRS4 ortholog (*light blue*).

We next sought to identify and analyze microsyntenic blocks of the *COL4* genes to gain additional evidence for a genetic linkage between *COL4A*⟨1|2⟩ and *COL4A*⟨3|4⟩ and a linkage between *COL4A*⟨1|2⟩ or *COL4A*⟨3|4⟩ and the *COL4A*⟨5|6⟩ gene pair. A microsyntenic block containing each of the three vertebrate *COL4* gene pairs and the IRS gene family was previously noted, wherein an IRS paralog exists in close proximity to each of the three *COL4* gene-pair paralogs (Fig. 4) (54). This finding prompted us to further identify the order and conservation of genes neighboring the *COL4* gene pairs as an approach for determining genetic linkages.

### Microsynteny of *COL4A⟨1|2⟩* gene pair in vertebrate evolution

**Figure 5:**
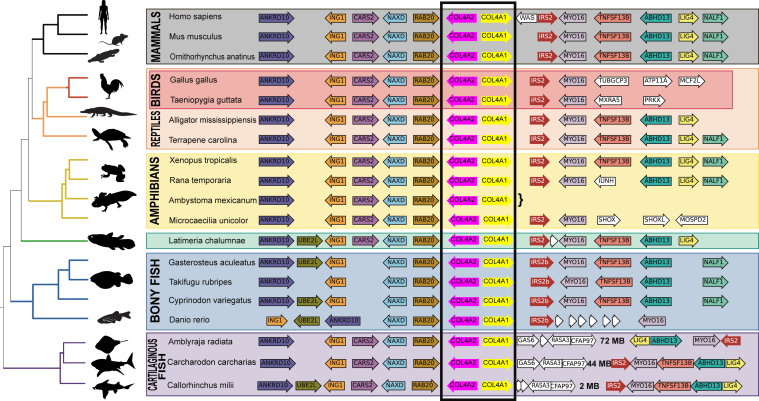
**Vertebrate synteny of the COL4A⟨1|2⟩ gene-pair locus.** Genome sequences from selected and diverse jawed vertebrates were examined surrounding the *COL4A*⟨1|2⟩ gene pair, and protein-coding genes and their orientations were identified. These are indicated as rows next to their gene names for each species. The standard National Center for Biotechnology Information phylogenetic tree is indicated to the *right* of each species. The axolotl genome is very large, and the contig containing *COL4A2* is shown; *curly brace* (}) marks the boundaries of the contig where the annotated chromosome is likely misassembled. Genes indicated in *white* are either low-confidence gene annotations or genes that are not syntenic to other vertebrates. Note that there is a chromosome inversion in the chondrichthyes fishes. This places the *IRS* gene distal to the *COL4A*⟨1|2⟩ locus; the genes immediately adjacent to *COL4A*⟨1|2⟩ are shown as are the syntenic genes; however, intervening genes are omitted for clarity (omitted genes are indicated with the *ellipses*). The names of genes are based on orthology groups identified through blastp homology to the human genome annotation.

We identified the synteny for the *COL4A*⟨1|2⟩ gene pair in diverse species (Fig. 5) and found an extensive conservation of neighboring gene order. For example, in all mammals examined, the five genes on either side of the *COL4A*⟨1|2⟩ gene pair are absolutely conserved in order and orientation. Reptiles have near identical syntenic gene order, whereas birds have a chromosomal rearrangement 5′ of the *MYO16* locus. We next investigated the *COL4A*⟨1|2⟩ locus in amphibia. Extant amphibia consist of three orders: Salienta (frogs and toads), Caudata (salamanders and newts), and Caecilians. Notably, amphibian genomes tend to be very large, making genome assembly and, thus, microsyntenic block analysis difficult because of contig fragmentation. In the salamander genome, the *COL4A*⟨1|2⟩ containing contig is small, limiting microsynteny analysis; however, in the conserved synteny of the *COL4A*⟨1|2⟩ locus to other vertebrates is clear. The oldest extant group of tetrapods with a well-described genome is the Coelacanth whose genome also shows conserved microsynteny, essentially identical to that found in mammals. Thus, in tetrapods, the *COL4A*⟨1|2⟩ microsyntenic block is highly conserved.

We next investigated whether this synteny was conserved within fishes (Fig. 5). Teleosts, which exhibit extraordinary diversity in ecology, behavior, and morphology, may show more diversity, which may be more revelatory of the origins of this synteny group; however, we found that in diverse teleost groups, the core of the *COL4A*⟨1|2⟩ microsyntenic region was well conserved. In cartilaginous fishes, there is a significant deviation from the microsynteny: the region 3′ of *COL4A1* diverged from that observed in other vertebrate groups. However, the microsyntenic block to the region 3′ of *COL4A1* occurs nearby on the same chromosome, which suggest that through a chromosomal break and inversion, the microsynteny was lost but synteny was retained. This result suggests that a chromosomal break and inversion proximal to the *COL4A1* 3′ region occurred distinguishing the Chondrichthyes (cartilaginous fishes) and Osteichthyes (bony fishes and their descendants). Together, these findings reveal a highly conserved syntenic block of genes containing the *COL4A*⟨1|2⟩ gene pair, which was maintained throughout jawed vertebrate evolution.

### Microsyntenies of *COL4A⟨3|4⟩* and *COL4A⟨5|6⟩* gene pairs in vertebrate evolution

**Figure 6:**
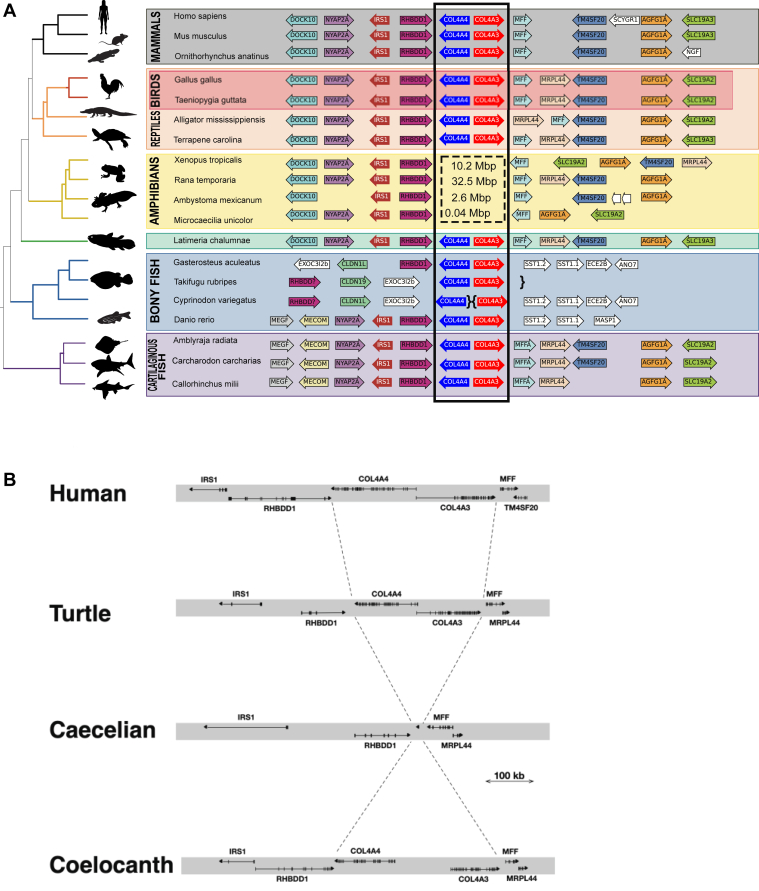
**Vertebrate synteny of the *COL4A*⟨3|4⟩ gene-pair locus, and deletion of the gene pair in amphibians.***A*, genome sequences from selected and diverse jawed vertebrates were examined surrounding the *COL4A*⟨3|4⟩ gene pair, and protein-coding genes and their orientations were identified as for Figure 6. In zebrafish, the genes found downstream of *COL4A3* in other vertebrates, including *SLC19A3*, *AGFG1*, *MRPL4*, and *MFF*, are found upstream of *COL4A1*, consistent with a chromosome inversion. The absence of the *COL4A*⟨3|4⟩ locus in amphibians is indicated with the *dashed box*. The size of the chromosome fragment in each species replacing the *COL4A*⟨3|4⟩ locus is indicated in the *box*. In the Caecilian genome, a single gene encoded by a single exon encoding a kinase similar to *TSSK6* in humans was found in this intergenic region, whereas in Axolotl, no genes were observed in the intergenic gap. In the two frog species, multiple genes were found in the intergenic region. *B*, the exon–intron structure of selected jawed vertebrate genomes reveals the precise deletion of *COL4A*⟨3|4⟩ in the Caecelian genome: neither neighboring genes are affected save that *MFF* is inverted in some amphibians.

We identified a microsyntenic region for the *COL4A*⟨3|4⟩ gene pair and found extensive conservation throughout jawed vertebrate (Gnathostome) evolution (Fig. 6*A*). Within the tetrapod lineage in mammalian, reptilian, and avian genomes, the genes flanking *COL4A*⟨3|4⟩ are well conserved forming a microsyntenic block (Fig. 6*A*). This conservation is retained in the Coelacanth and cartilaginous fishes, though is less conserved in teleosts. Strikingly, the IRS paralog associated with *COL4A*⟨3|4⟩ is found in a different orientation and gene order throughout this lineage compared with the *COL4A* gene pair when compared with *COL4A*⟨1|2⟩. While *IRS2* is found downstream of *COL4A1*, *IRS1* is found downstream of *COL4A4* and the *RHBDD1* gene intervenes between *COL4A4* and *IRS1*.

Surprisingly, the syntenic analyses revealed that the *COL4A*⟨3|4⟩ gene pair was deleted in amphibian genomes (Fig. 6*A*). The deletion was precise, retaining the neighboring genes, *IRS1* and *RHBDD1*, which are 3′ to *COL4A4* in other vertebrates and *MFF* and *MRPL44*, which are 3′ to *COL4A3* in other vertebrates (Fig. 6*B*). The caecilian genome has a novel single exon protein coding transcript that was detected in the small gap between *RHBDD1* and *MFF*, but there is no clear insertion of functional DNA at this locus compared with those found in both more basal tetrapods (Coelacanth) and more advanced tetrapods (reptiles and mammals). In contrast, larger insertions are observed at the locus in other amphibian classes. Notably, *COL4A*⟨3|4⟩ gene pair was absent in other Amphibian orders. Notably, this precise deletion of the *COL4A*⟨3|4⟩ gene pair occurs in species that otherwise have quite large and expanded genomes. Parenthetically, this finding of a naturally occurring double gene knockout provided a strategy in a companion article (25) to gain insights into critical molecular features the Col-IV**^α345^** scaffold that confer function to the GBM.

**Figure 7:**
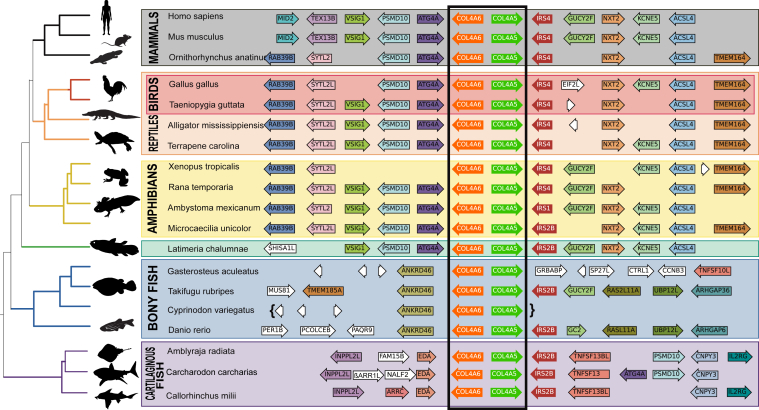
**Vertebrate synteny of the COL4A⟨5|6⟩ gene-pair locus.** Genome sequences from selected and diverse jawed vertebrates were examined surrounding the *COL4A*⟨5|6⟩ gene pair, and protein-coding genes and their orientations were identified as for Figure 2. Note that the synteny is not well maintained in the teleost (*blue*) lineage perhaps because of the teleostean-specific whole-genome duplication. Note the presence of the *ANKRD46* gene adjacent to *COL4A6* in the teleost lineage, which is paralogous to *ANKRD10* found downstream of *COL4A2*, suggesting that these genes share a common ancestry, which was duplicated together with the *COL4A* gene pair generating *COL4A*⟨5|6⟩.

We next identified the microsyntenic block containing the *COL4A*⟨5|6⟩ gene pair. The *COL4A*⟨5|6⟩ loci in mammalian genomes differs distinctly from both *COL4A*⟨1|2⟩ and *COL4A*⟨3|4⟩, which have microsyntenic blocks that are nearly identical (Fig. 7). The *IRS4* locus is found downstream and encoded by the opposite strand from *COL4A5* compared with *IRS* and *COL4A1*, suggesting that the duplicated syntenic block, which generated these paralogies, underwent rapid rearrangements following duplication. The decreased conservation of the syntenic regions surrounding *COL4A*⟨5|6⟩ is most pronounced in the Teleosts, which have quite reduced extents of synteny conservation. Collectively, these results reveal that jawed vertebrates have a well-conserved syntenic block surrounding the *COL4A*⟨1|2⟩, *COL4A*⟨3|4⟩, and *COL4A*⟨5|6⟩ gene pairs.

### Analysis of microsynteny for lineage of *COL4* gene pairs in deuterostomes

**Figure 8:**
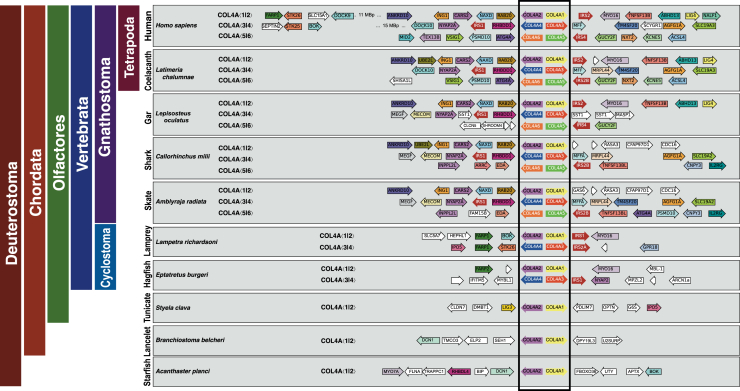
***COL4A* gene locus evolution in the deuterostomes.** The microsyntenic regions at each *COL4A*⟨1|2⟩ gene pair (*boxed*) from selected species are shown. In deuterostome, the first species to have synteny beyond the *COL4A*⟨1|2⟩ gene pair is the hagfish, which is also the first species to demonstrate duplication of the *COL4A*⟨1|2⟩ gene pair into *COL4A*⟨3|4⟩. Lampreys demonstrate increased regional synteny as well as two *COL4A* gene pairs. Note the presence of paralogs of *FARP*, *BOK*, *IPO5*, and *STK25* in lampreys and hagfish that is retained in mammals, albeit at a more distal position on the linkage group. Gnathostomes, except amphibia, show all three *COL4A* gene pairs.

We next sought to analyze the microsyntenic blocks to gain any corroborative evidence for *COL4A*⟨1|2⟩ to be the progenitor for *COL4A*⟨3|4⟩ and to determine whether *COL4A*⟨1|2⟩ or *COL4A*⟨3|4⟩ is the progenitor of the *COL4A*⟨5|6⟩ gene pair. Early deuterostomes (tunicate, lancelet, and starfish) have only a single pair of *COL4A* genes, corresponding to *COL4A*⟨1|2⟩ gene pair and without conserved synteny (Fig. 8). Synteny of *COL4A* genes first appears in hagfish and lamprey, each of which have two *COL4* gene pairs, *COL4A*⟨1|2⟩ and *COL4A*⟨3|4⟩. In lamprey, both *COL4* gene pairs are adjacent to an *IRS* paralog, as seen in jawed vertebrates. However, in hagfish, one *COL4* gene pair has an *IRS* paralog adjacent, whereas the other does not. In hagfish, there is an additional synteny conservation in the form of the *MYO16* and *NYAP2* genes, which lie adjacent to each *IRS* paralog and downstream from the *COL4A* gene pair. This is a highly similar gene arrangement to those found in the *COL4A*⟨1|2⟩ and *COL4A*⟨3|4⟩ synteny groups. This result strongly suggests an evolutionary orthology between the cyclostome *COL4* gene pair adjacent to *MYO16* and the vertebrate *COL4A*⟨1|2⟩ as well as a similar relationship between the cyclostome *COL4* gene pair, which is adjacent to *NYAP2* and the vertebrate *COL4A*⟨3|4⟩. Between the last common ancestor of cyclostomes and jawed vertebrates, the *COL4A*⟨3|4⟩ gene pair became inverted, moving the *NYAP2-IRS* genes downstream from *COL4A4* rather than downstream from *COL4A3* as seen in the cyclostomes. We note also the presence of the genes *SLC5A7*, *FARP2*, and *BOK* downstream of the lamprey *COL4A2* gene and *STK26*, *FARP1*, and *IPO5* downstream of the *COL4A4* gene. Paralogs of these genes also appear downstream of *COL4A2* and *COL4A4* in the vertebrate genome, albeit several Mbp further downstream of the *COL4A* loci.

Collectively, the syntenic analyses reveal that cyclostomes, hagfish and lamprey, have two pairs of *COL4* genes, corresponding to *COL4A*⟨1|2⟩ and *COL4A*⟨3|4⟩ gene pairs in vertebrate, and devoid of *COL4A*⟨5|6⟩. In contrast, shark has six *COL4* genes that correspond to vertebrate *COL4A*⟨1|2⟩, *COL4A*⟨3|4⟩, and *COL4A*⟨5|6⟩ gene pairs. Thus, the *COL4A*⟨1|2⟩ gene pair duplicated first in cyclostomes and evolved into the *COL4A*⟨3|4⟩ gene pair. Secondarily, in shark, a duplication of the *COL4A*⟨1|2⟩ or the *COL4A*⟨3|4⟩ gene pair gave rise to *COL4A*⟨5|6⟩ in shark; the identity of which pair was not apparent from the microsynteny.

### Analysis of amino acid sequences for lineage of *COL4* gene pairs

We sought to obtain further evidence for lineage of gene duplications from a phylogenetic analysis of the amino acid sequences of the six α-chains, which coemerged in shark. The analysis for full-length α-chains across metazoans (Fig. S2) and their cognate NC1 domains (Fig. S3) reveal that shark α3 and α5 chains most closely related to the α1 chain. In contrast, the shark α4 and α6 chains related to the α2 chain. These findings suggest that *COL4A*⟨1|2⟩ is the progenitor for both the *COL4A*⟨3|4⟩ and *COL4A*⟨5|6⟩ gene pairs.

### Expression of *COL4* gene pairs in hagfish and shark kidneys

The conservation of *COL4* gene pairs across metazoans posits the question of whether the *COL4A*⟨1|2⟩ and *COL4A*⟨3|4⟩ gene pairs in hagfish and the *COL4A*⟨1|2⟩, *COL4A*⟨3|4⟩, and *COL4A*⟨5|6⟩ gene pairs in shark are expressed and incorporated into the kidney. At the time this study began, genomic data for these species were unavailable. Therefore, to probe this question, we isolated total RNA from both hagfish and dogfish (shark) kidneys and performed next-generation RNA-Seq. *De novo* assembly of the dogfish transcriptome was performed and used to design specific primers to PCR *COL4A* transcripts, which were subsequently sequenced to confirm the transcriptomics results. We found that at least three *COL4A* transcripts (corresponding at the protein level to the Col-IV α1, α2, and α3 or α4 chains) were expressed in hagfish kidney, whereas all six transcripts (corresponding to the α1–α6 chains) were expressed in dogfish kidney (Supporting information).

**Figure 9:**
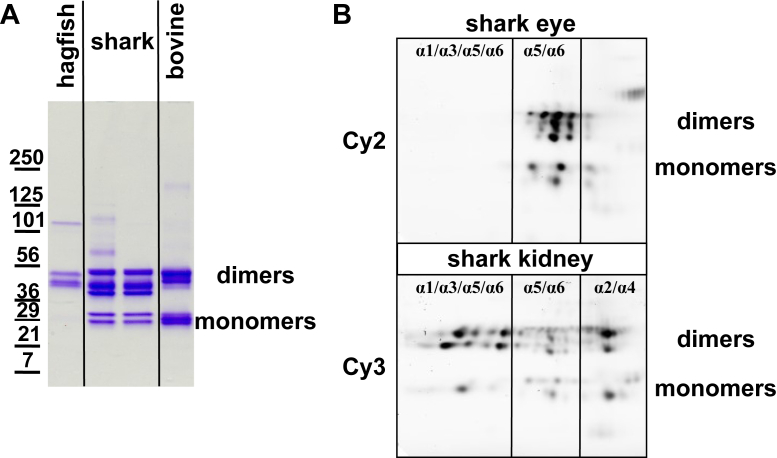
**Tissue-specific collagen IV (Col-IV) chain composition.***A*, SDS-PAGE of purified noncollagenous (NC1) domains of Col-IV derived from hagfish kidney, shark ocular lens and kidney, and bovine kidney. The shark and bovine NC1 domains run as both uncrosslinked monomers and crosslinked dimers, whereas the hagfish protein does not have detectable monomers. The shark dimer band runs as a set of three bands likely reflecting scaffolds of different compositions. *B*, fluorescent images of the 2D-NEPHGE gel loaded with purified NC1 domains from the shark ocular lens (Cy2) and kidney (Cy3). Interestingly, shark ocular lens consists primarily of α5 and α6, whereas shark kidney has all chains present. 2D-NEPHGE, two-dimensional nonequilibrium pH gel electrophoresis.

We determined whether the Col-IV α1–α6 chains were incorporated into hagfish and dogfish kidney in the form of supramolecular scaffolds. We used the well-established method of characterization of the noncollagenous NC1 hexamers from the scaffolds, as direct evidence for scaffold organization (55). NC1 hexamers were excised by collagenase digestion, purified by size-exclusion chromatography, and characterized by SDS-PAGE. The electrophoresis patterns were similar to that of mammalian hexamer by the presence of NC1 dimer and monomer subunits, revealing scaffold expression in both hagfish and shark (29, 56) (Fig. 9*A*).

To determine which α-chains were incorporated into shark kidney, we identified the α-chain origin of the NC1 hexamer subunits. The analysis was performed by using two-dimensional nonequilibrium pH gel electrophoresis (2D-NEPHGE) on kidney and lens tissues. Kidney hexamer was labeled with the Cy3 fluorescent dye and lens hexamer with Cy2. The fluorescent-labeled hexamers were mixed and resolved by 2D-NEPHGE (Fig. 9*B*). The lens and kidney patterns were distinct, revealing tissue-specific differences in the expression of α-chains. Individual spots on the gels were excised and analyzed with high-resolution LC–MS/MS. A custom dogfish protein database, based on the transcriptomics data, was used to identify α-chain identity of each spot (Figs. S4, S9). The results show that all six Col-IV α-chains (α1–α6) were expressed in shark kidney whereas only the α5 and α6 chains in lens. The scaffold compositions of these multiple α-chains remain unknown, but they do not include the Col-IV**^α345^** scaffold in shark kidney as described in a companion article (25).

### Evolutionary changes in the number of cysteine codons in the *COL4A⟨3|4⟩* gene pair

In prior studies, we found that Col-IV**^α345^**, the principal component of GBM, is highly crosslinked by disulfide bonds in comparison to the Col-IV**^α121^** scaffold (57). Specifically, the number of cysteine residues is very large in the α3 and α4 chains, in comparison to the other Col-IV α1, α2, α5, and α6 chains. We speculated that disulfide crosslinks are a key structural feature that confers mechanical strength to the GBM as well as to protect against proteolysis (57). To gain insights into the role of cysteine residues, we investigated the phylogenetic distribution of their codons in the *COL4* gene pairs.

**Figure 10:**
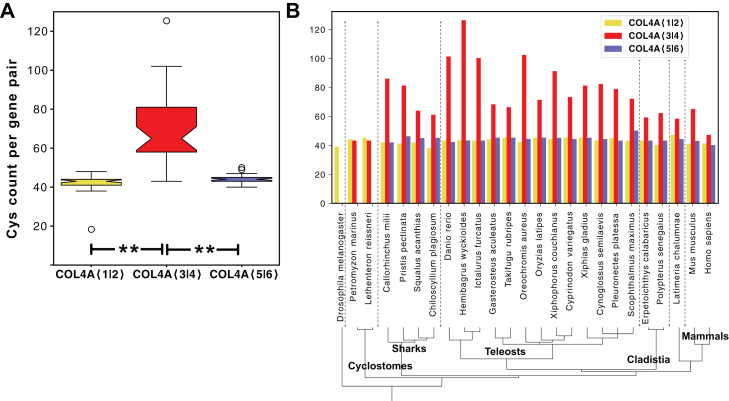
**Cysteine content is increased in *COL4A*⟨3|4⟩ gene pairs.***A*, both *COL4A*⟨1|2⟩ and *COL4A⟨*5|6⟩ encode similar numbers of cysteine residues per gene pair, whereas the *COL4A*⟨3|4⟩ gene pair encodes an increased number of cysteine residues in diverse vertebrates. *B*, comparison of the cysteine codon count per gene pair in diverse vertebrate species is not correlated with clade.

**Figure 11:**
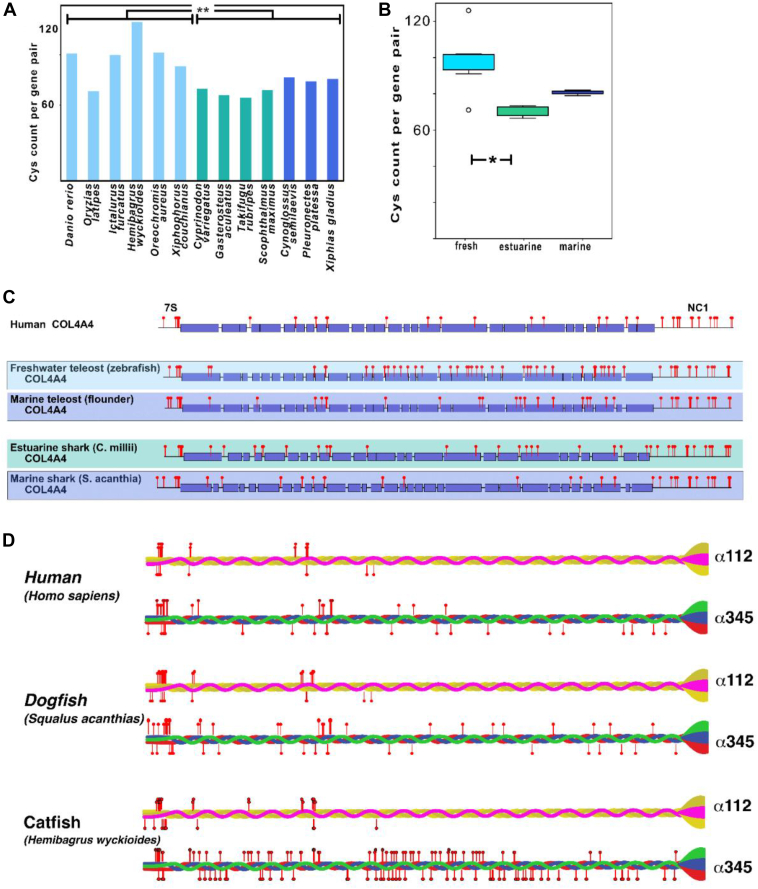
**Cysteine content in *COL4A*⟨3|4⟩ gene pair varies by animal habitat.***A*, cysteine count in teleosts found in different osmolarity habitats (freshwater, *pale blue*; estuarine, *teal*; and marine, *dark blue*). Freshwater fishes have increased cysteine content compared with marine and estuarine fishes. *B*, cysteine count in diverse fishes including teleosts and cartilaginous fishes confirming the increased cysteine counts in freshwater fishes generally. *C*, map of the cysteines (*red pins*) on the *COL4A4* reading frame from humans and diverse fishes. Collagen-encoding domains are *colored boxes* and noncollagenous domains are indicated by *lines*, 7S and noncollagenous (NC1) domains are indicated at the N termini and C termini, respectively. *D*, cysteines mapped to the protomer for human, estuarine shark, and freshwater catfish Col-IV**^α345^**. Odd numbered *COL4A* genes are mapped above, and even numbered *COL4A* genes are mapped below the protomer schematic. Cysteines in the NC1 domain are known to form disulfide bonds that stabilize the domain and are not shown (80, 81).

We identified a significant increase in the cysteine codons in the *COL4A*⟨3|4⟩ gene pair, compared with *COL4A*⟨1|2⟩ and *COL4A*⟨5|6⟩ (Fig. 10*A*), and also a very large variation in numbers of codons among diverse species (Fig. 10*B*). The variance occurred both within and between clades (Fig. 11*A*) and specifically when vertebrates were classified by their environment rather than their clade (Fig. 11*B*). A statistically significant difference was identified within the teleost group when they were classified by the salinity of their environment (Fig. 11*B*). Fish in freshwater environments have increased cysteine content compared with those found in estuarine and marine environments. Notably however, this difference in cysteine content is not present in the cyclostomes (Fig. 10*A*).

We next determined whether there was a pattern to the change in cysteine abundance that may relate to function. When the cysteine codons were plotted on the predicted open reading frame of the *COL4A4* gene, we found that the variable cysteine residues were scattered throughout the region of the collagenous domain (Fig. 11*C*, and Fig. S5). The increase in cysteine content was most notable in the C-terminal half of the collagenous domain. At the protein level, the abundance of cysteine residues is a distinguishing feature of the triple-helical protomers that assemble into the Col-IV**^α345^** scaffold, in comparison with the Col-IV**^α121^** scaffold (Fig. 1*D*). Notably, catfish has the largest number of cysteine residues in a Col-IV**^α345^** protomer, suggesting a key adaptation enabling GBM function in freshwater animals.

## Discussion

In our previous work, we found that Col-IV is a primordial component of basement membranes that enabled the assembly of a fundamental architectural unit for the genesis and evolution of multicellular tissues (1). Also, we found that the structural domains of vertebrate Col-IV protomers, described in Figure 2, are conserved across metazoans. Moreover, the pairwise arrangement of *COL4* genes also appeared to be conserved, based on the analysis of a limited number of species, but the phylogeny and identity of gene pairs remain unknown.

**Figure 12:**
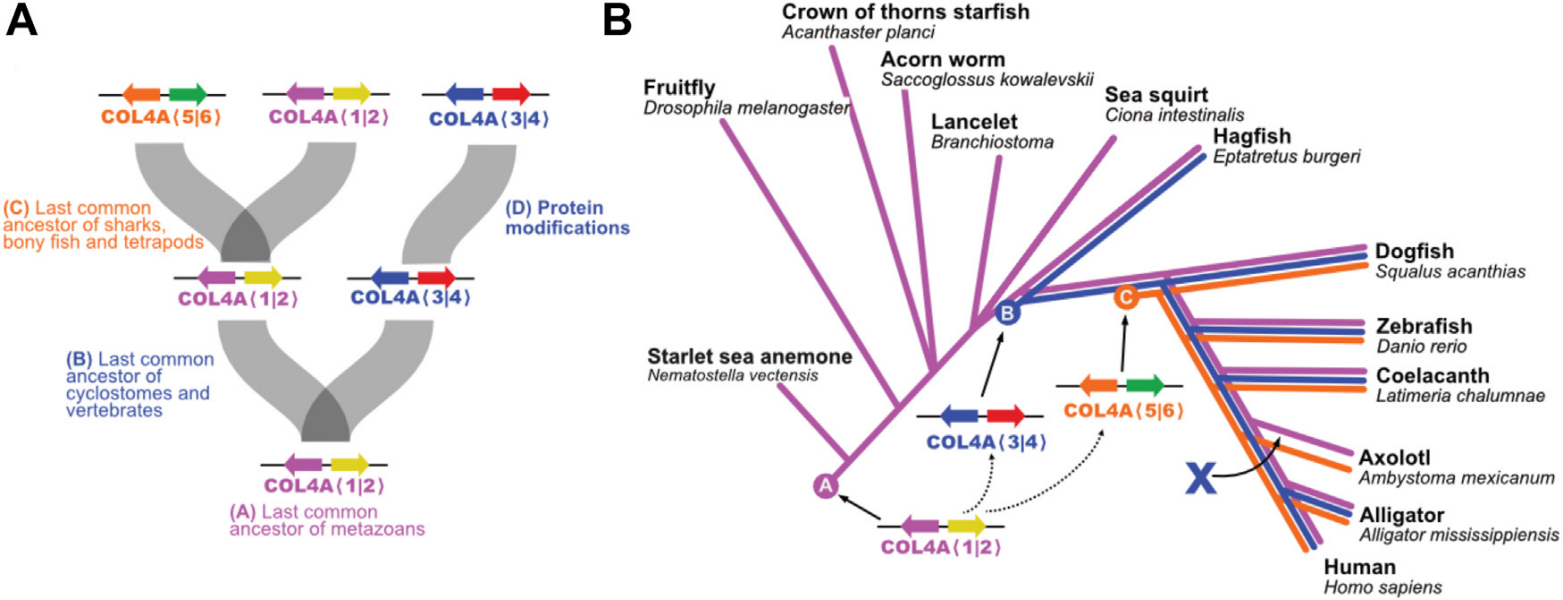
**COL4A gene evolutionary history in multicellular animals.***A*, hypothesized gene duplication events resulting in generation of the *COL4A*⟨3|4⟩ and *COL4A*⟨5|6⟩ with the apparent last common ancestors at each step indicated. *B*, highly schematic cladogram of each duplication event (indicated by the letters A, B, and C) and each gene pair indicated by the color of the gene pair. The X indicates the loss of the *COL4A*⟨3|4⟩ gene pair observed in extant amphibians.

**Figure 13:**
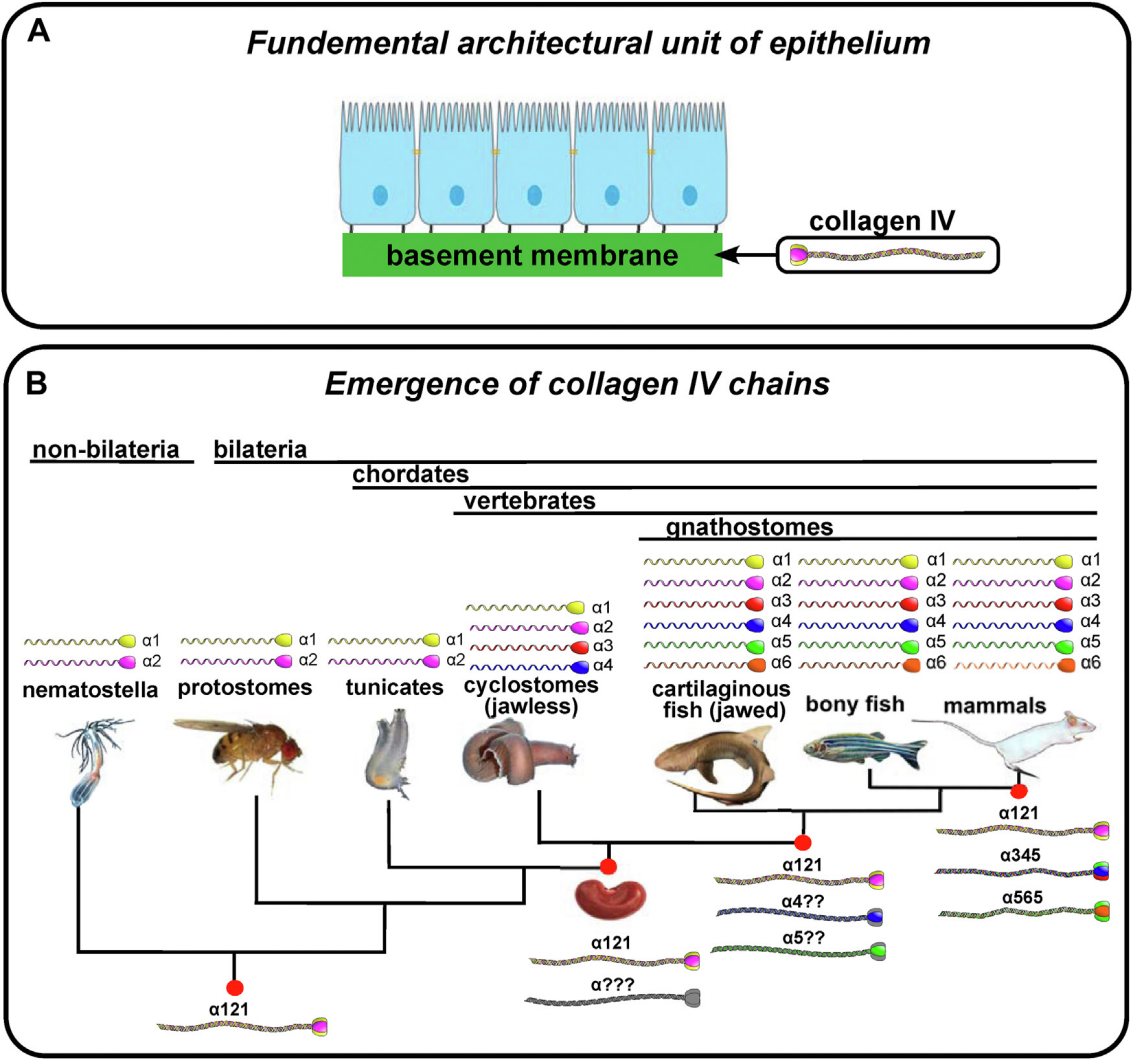
**Evolutionary emergence of collagen IV (Col-IV) α3 through α6 chains coincides with kidney appearance followed by GBM morphological transition upon emergence of Col-IV^α345^.***A*, this architectural unit of epithelia tissues is characterized by a layer of apical/basal-polarized cells that are laterally connected by tight junctions between plasma membranes, which are basally anchored *via* integrin receptors embedded in plasma membranes to a basement membrane suprascaffold. In turn, this architectural unit served as the building block that enabled the formation and evolution of epithelial tissues, the ever-increasing complexity and size of organisms, and for the expansion and diversity of the animal kingdom. Col-IV triple helical protomers, a principal component of basement membranes, was a primordial innovation in early metazoan evolution that enabled the transition to multicellularity and the evolution of epithelial tissues in metazoa (1). *B*, genome duplication events led to appearance of *COL4A*⟨3|4⟩ and then *COL4A*⟨5|6⟩. Animals have two or more chains of Col-IV generating the Col-IV**^α121^** scaffold. In cyclostomes, the α3 and α4 chains first appeared; and α5 and α6 appeared later in cartilaginous and bony fishes. Emergence of *COL4A*⟨3|4⟩ coincides with the appearance of the glomerulus, which generates a high volume of filtrate, which is processed by the nephron tubule. However, the mammalian GBM morphology and ultrafilter function are only found in vertebrates that evolved to have Col-IV**^α345^** scaffold (25). GBM, glomerular basement membrane.

Here, we sought to extend these phylogenetic analyses to determine the emergence and genetic lineage of the *COL4* gene family, with an emphasis on those encoding the Col-IV**^α345^** scaffold. We anticipated this strategy would provide insights into structure–function relationships of the Col-IV**^α345^** scaffold that enabled the GBM to function as an ultrafilter of proteins. We found that the *COL4A*⟨1|2⟩ gene pair emerged in basal Ctenophores and Cnidaria phyla and is highly conserved across metazoans (Fig. 12). The *COL4A*⟨1|2⟩ duplicated and arose as the progenitor to the *COL4A*⟨3|4⟩ gene pair in cyclostomes, coinciding with emergence of kidney GBM, and expressed and conserved in jawed vertebrates, except for amphibians, and a second duplication as the progenitor to the *COL4A*⟨5|6⟩ gene pair and conserved in jawed vertebrates. These findings revealed the genetic emergence and expression of the Col-IV α1 and α2 chains that assemble into the Col-IV**^α121^** scaffold and incorporate ubiquitously in metazoan basement membranes. Moreover, Col-IV**^α121^** is the progenitor scaffold that evolved into vertebrate Col-IV**^α345^** scaffold and incorporated mainly in the GBM of mammals (Fig. 13). In a companion article, we found that the emergence of the Col-IV**^α345^** scaffold enabled the assembly of a compact GBM that functions as the primary ultrafilter of proteins in mammals (25).

**Figure 14:**
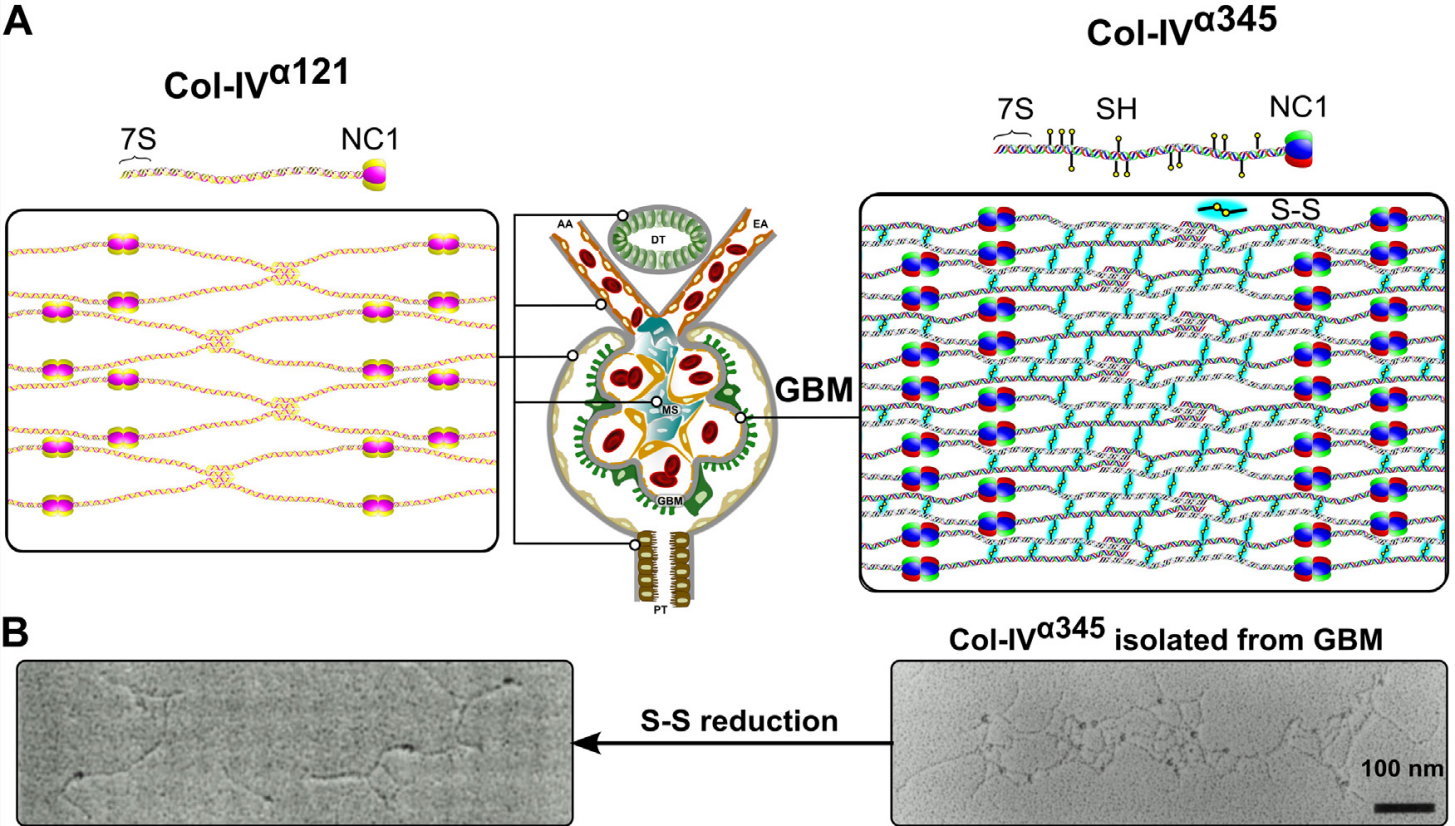
**Reinforcement of collagen IV (Col-IV)^α345^ scaffolds in the mammalian GBM by disulfide-mediated crosslinks between protomers.***A*, the Col-IV**^α121^** scaffold schematically represented on the *left* is a component of the basement membranes surrounding kidney tubules (PT), efferent and afferent arterioles (EA and AA), and Bowman’s capsule. It is also found in the mesangial space (MS). The Col-IV**^α345^** scaffold shown on the *right* is the principal component of the GBM. This scaffold is reinforced by multiple disulfide bonds (S-S bonds highlighted in *cyan*) between protomers. *B*, rotary shadowing electron microscopy images of Col-IV**^α345^** scaffold, isolated from bovine kidney glomeruli (*right*), and the same scaffold after reduction of the disulfide bonds (*left*). Upon reduction of lateral disulfide crosslinks, the scaffold dissociates into protomers, which are dimerized through their noncollagenous (NC1) domains (57). GBM, glomerular basement membrane.

In prior studies, we found that Col-IV**^α345^**, the principal component of GBM, is highly crosslinked by disulfide bonds in comparison to the Col-IV**^α121^** scaffold (Fig. 14) (57). We speculated that disulfide crosslinks are a key structural feature that confers mechanical strength to the GBM as well as to protect again proteolysis. Intriguingly, in the present study, we found that the Col-IV**^α345^** scaffold has an increased number of cysteine residues in comparison to Col-IV**^α121^**, which mediate disulfide crosslinks between protomers in lateral associations and supercoils (11), and which vary in number with the osmolarity of the environment. The GBM is the only known basement membrane in which there is bulk flow of liquid rather than diffusion across the membrane. This bulk flow, associated with glomerular ultrafiltration, places high hydrostatic pressure (58) on the GBM. In freshwater teleost, such as catfish and zebrafish living in low osmolarity environments, there is high liquid flux across the GBM, compared with marine animals in high saline environments. Thus, the increase in cysteine content likely confers additional mechanical strength to GBM to withstand high hydrostatic pressure. This environmental adaptation in disulfide crosslinking pinpoints a role for Col-IV**^α345^** scaffold in the GBM *versus* Col-IV**^α121^**, wherein Col-IV**^α345^** having an increased number of crosslinks enabled the assembly of a compact GBM that withstands the high hydrostatic pressure in mammals (Fig. 14) (25).

It is noteworthy that the GBM of terrestrial amphibians is devoid of the Col-IV**^α345^** scaffold (25). The terrestrial environment, rather than freshwater, required quite distinct water retention mechanisms, suggesting that Col-IV**^α345^** scaffold was counterproductive. Unlike most vertebrates in which the glomerular filtrate drains from Bowman’s space into the proximal tubule, the amphibian glomerular filtrate drains into the coelom (59) and the coelomic fluid then passes into the nephron tubule. Such an anatomical arrangement increases the surface area available for protein and electrolyte absorption, perhaps obviating the need for high fluid flow across the GBM and the necessity for a compact GBM. Instead, amphibians adapted the slit diaphragm as the primary ultrafilter of proteins.

## Experimental procedures

### Next-generation RNA-Seq

Transcriptomes used in this study were sequenced at the Vanderbilt Technologies for Advanced Genomics Core Facility (VANTAGE). The Illumina TruSeq mRNA Sample Preparation Kit was used to convert the mRNA in 100 ng of total RNA into a library of template molecules suitable for subsequent cluster generation and sequencing on the Illumina HiSeq 2500 using the rapid run setting. The pipeline established in VANTAGE was followed and is briefly described later. The first step was a quality check of the input total RNA by running an aliquot on the Agilent Bioanalyzer to confirm RNA integrity. The Qubit RNA fluorometry assay was used to measure sample concentrations. The input-to-library prep was 100 ng of total RNA (2 ng/μl). The poly-A containing mRNA molecules were concentrated using poly-T oligo-attached magnetic beads. Following purification, the eluted poly(A) RNA was cleaved into small fragments of 120 to 210 base pair (bp) using divalent cations under elevated temperature. The cleaved RNA fragments were copied into first-strand complementary DNA (cDNA) using SuperScript II reverse transcriptase and random primers. This step was followed by second-strand cDNA synthesis using DNA Polymerase I and RNase H treatment. The cDNA fragments then went through an end repair process, the addition of a single “A” base, and then ligation of the Illumina multiplexing adapters. The products were then purified and enriched with PCR to create the final cDNA sequencing library. The cDNA library then undergoes quality control by running on the Agilent Bioanalyzer HS DNA assay to confirm the final library size and on the Agilent Mx3005P qPCR machine using the KAPA Illumina library quantification kit to determine concentration. A 2 nM stock was created, and samples were pooled by molarity for multiplexing. From the pool, 12 pmoles were loaded into each well for the flow cell on the Illumina cBot for cluster generation. The flow cell was then loaded onto the Illumina HiSeq 2500 utilizing v3 chemistry and HTA 1.8. The raw sequencing reads were processed through CASAVA-1.8.2 for FASTQ conversion and demultiplexing. The Illumina chastity filter was used, and only the PF (passfilter) reads are retained for further analysis. *De novo* assembly of transcriptomes was performed using Velvet/Oases and Trinity software packages with default settings (60, 61, 62). The accuracy of *de novo* assembly was checked in a parallel next-generation RNA-Seq experiment using RNA from mouse PFHR9 cells. *De novo* assembled transcripts were used to generate BLAST databases to search for Col-IV hits using tblastn (63) with e-value cutoff set to 10^−15^. Multiple sequence alignments and conserved domain searches were performed with the Geneious v5-6 software (Biomatters).

### Cloning of hagfish and dogfish *COL4A*

To confirm accuracy of hagfish and dogfish COL4A, cDNA sequences obtained from RNA-Seq–based sequences, we performed a series of RT–PCR cloning experiments using primers designed to NGS-detected COL4A candidates. RNA was prepared using the QIAGEN One-Step RT–PCR Kit, and PCR products were cloned using the QIAGEN PCR Cloning Kit.

### Isolation, purification, and analysis of Col-IV NC1 hexamers

Tissues were frozen in liquid nitrogen, pulverized in a mortar and pestle, and then homogenized in 2.0 ml g^−1^ digestion buffer and 0.1 mg ml^−1^ Worthington Biochemical bacterial collagenase and allowed to digest at 37°C, with spinning for 24 h. LC purification of solubilized NC1 varied by species based on protein yield. All ctenophore NC1s were purified by gel-exclusion chromatography (GE Superdex 200 10/300 GL). For reduction and alkylation of Col-IV NC1 hexamers, fractions containing high–molecular-weight complex from size-exclusion chromatography were concentrated by ultrafiltration and reduced in Tris-buffered saline buffer with various concentrations of DTT. After incubation for 30 min at 37°C, samples were alkylated with twofold molar excess of iodoacetamide for 30 min at room temperature in the dark. After mixing with SDS loading buffer, samples were heated for 5 min in a boiling water bath and analyzed by nonreducing SDS-PAGE. Collagenase-solubilized NC1 hexamers were analyzed by one-dimensional SDS-PAGE in 12% *bis*-acrylamide minicells with Tris–glycine–SDS running buffer. Sample buffer was 62. 5 mM Tris–HCl, pH 6.8, 2% SDS (w/v), 25% glycerol (w/v), and 0.01% bromophenol blue (w/v). Western blotting of SDS-dissociated NC1 hexamer was developed with JK-2, rat monoclonal antibody (kindly provided by Dr Yoshikazu Sado, Shigei Medical Research Institute. All Western blotting was done with Thermo-Scientific SuperSignal West Femto chemiluminescent substrate and digitally imaged with a Bio-Rad GelDoc system. Two-dimensional NEPHGE electrophoresis was performed according to the original protocol developed (64) with slight modifications developed in Hudson laboratory and used successfully to separate NC1 domains of Col-IV (65).

### Proteomics

All proteomics experiments were done at Vanderbilt’s MSRC Proteomics Core facility. Major protein spots from Coomassie-stained 2D-NEPHGE–separated dogfish Col-IV preparations isolated from various tissues were cut out of the gel, and proteins were identified and label-free quantified using MaxQuant (66, 67). The heatmap was created with heatmapper. ca based on label-free quantification data (68).

### Synteny analysis

Well-characterized genomes were selected from those appearing in the National Center for Biotechnology Information and Ensembl databases (69, 70) and the Axolotl genome (https://genome.axolotl-omics.org/cgi-bin/hgGateway; assembly ambMex 6. 0-DD), and *COL4* gene sequences were identified based on text and blastp (71) searches. Syntenic genes were then manually identified, and their orientation and order on the chromosome annotated and plotted with matplotlib (72). Protein domains were mapped to their ORFs using the Conserved Domain Database (73). The *COL4* gene phylogeny was derived from the National Center for Biotechnology Information Taxonomy (74, 75) and plotted using iTOL (76).

## Data availability

The shotgun transcriptome from wildtype adult *Myxine glutinosa* Atlantic hagfish has been deposited at DDBJ/EMBL/GenBank under the accession GKQO00000000. The version described in this article is the first version, GKQO01000000. The dogfish Transcriptome Shotgun Assembly project has been deposited at DDBJ/EMBL/GenBank under the accession GKOS00000000. The version described in this article is the first version, GKOS01000000. Proteomics data for shark have been submitted to the ProteomeXchange, projects PXD0441912 and PXD042111.

## Supporting information

This article contains supporting information.

## Conflict of interest

The authors declare that they have no conflicts of interest with the contents of this article.
