# Supplementary material for: Collagen IV of basement membranes: I. Origin and diversification of COL4 genes enabling metazoan multicellularity, evolution, and adaptation
Source: J Biol Chem. 2025 Apr 11;301(5):108496. doi: 10.1016/j.jbc.2025.108496 (PMC12141075; doi:10.1016/j.jbc.2025.108496)
Supplement: Supporting Information 2 [file mmc2.docx]

**
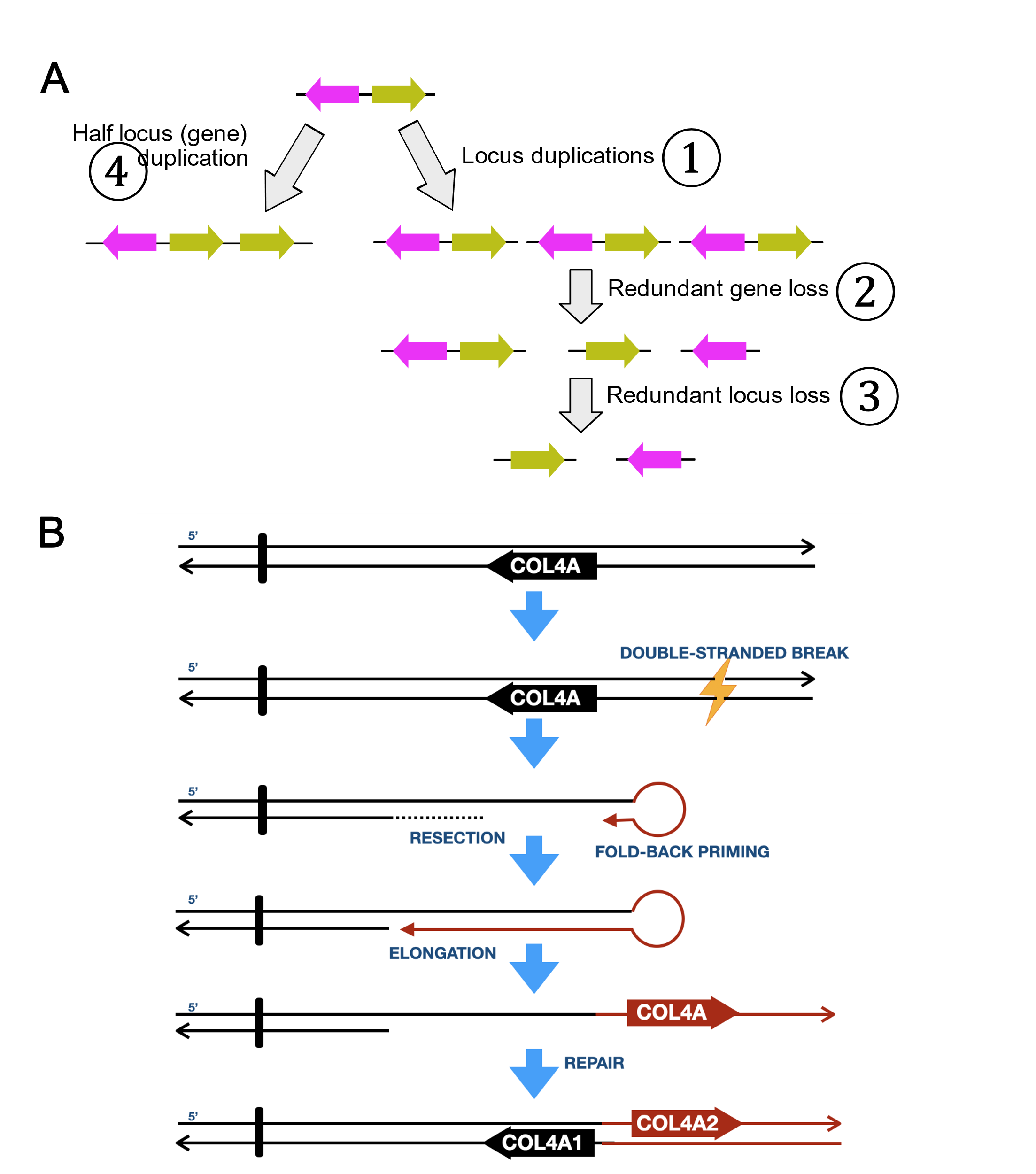
**

**Supporting figure 1. Hypothetical model for the generation of *COL4A* gene diversity**.

1. We propose that *COL4A* genes may be duplicated as pairs resulting in multiple copies of *COL4A* gene pairs as in *Adineta vega.* Individual *COL4A* genes may be deleted for some gene pairs as in *Capitella teleta*. While in other animals the original *COL4A*⟨1|2⟩ gene pair is lost leaving only the single *COL4A*1 and *COL4A*2 genes as is found *C. elegans*.
2. Proposed mechanisms for the generation of the *COL4A* gene pair in the head-to-head gene arrangement. The original *COL4A* gene is found likely in a telomeric position on the chromosome and its gene duplication is initiated through a double stranded break telomeric to the ancestral *COL4A* gene. Repair of the double stranded break proceeds through fold-back priming from the 3’ end of the top strand, resection and elongation through the *COL4A* gene (red strand), followed by resolution through multiple possible mechanisms. A similar model could occur in meiosis through creation of a dicentric chromatid that breaks during anaphase results in one gamete carrying a deletion for the ancestral *COL4A* gene and one carrying the duplicated *COL4A*⟨1|2⟩ gene pair.

**
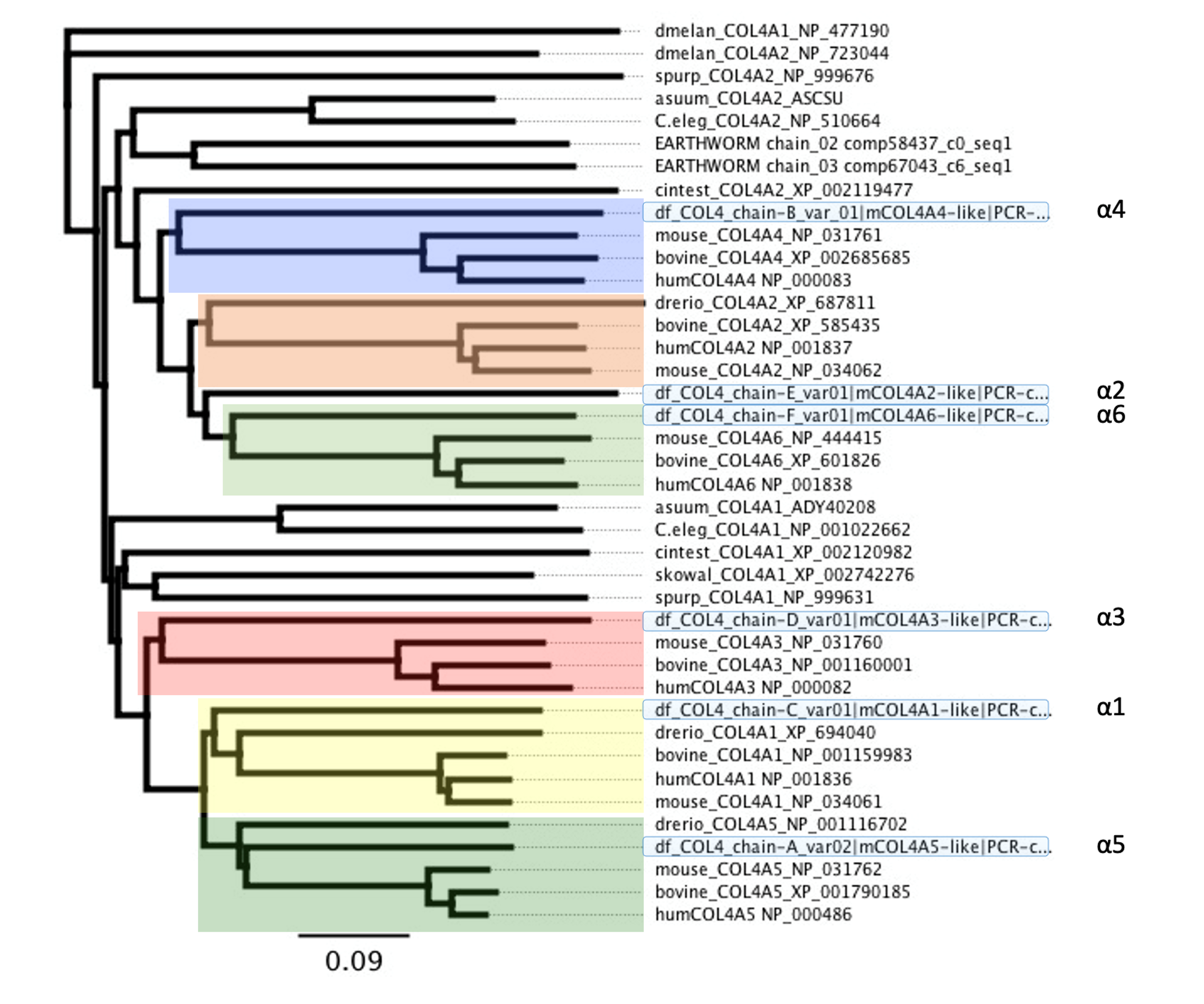
**

**Supporting figure 2. Phylogenetic comparison of Squalus acanthias collagen-IV full-length paralogs to representative metazoan species.**

Full-length sequences of *S. acanthias* (dogfish) were generated via RNASeq and de novo transcriptome assembly of dogfish basement membrane isolated from kidney, lens. Transcriptome sequences were confirmed by RT-qPCR. Assignment of sequenced dogfish chains was made based on known full length COL4 chains (a1-a6) from human, mouse, bovine, *Drosophila*, zebrafish, *C. elegans*, earthworm, *C. intestinalis*, *S. kowalevsky*, *S. purpuratis*. Phylogenetic tree is built using Neighbor-Joining method using Geneious Bioinformatic Software (https://www.geneious.com/).

**
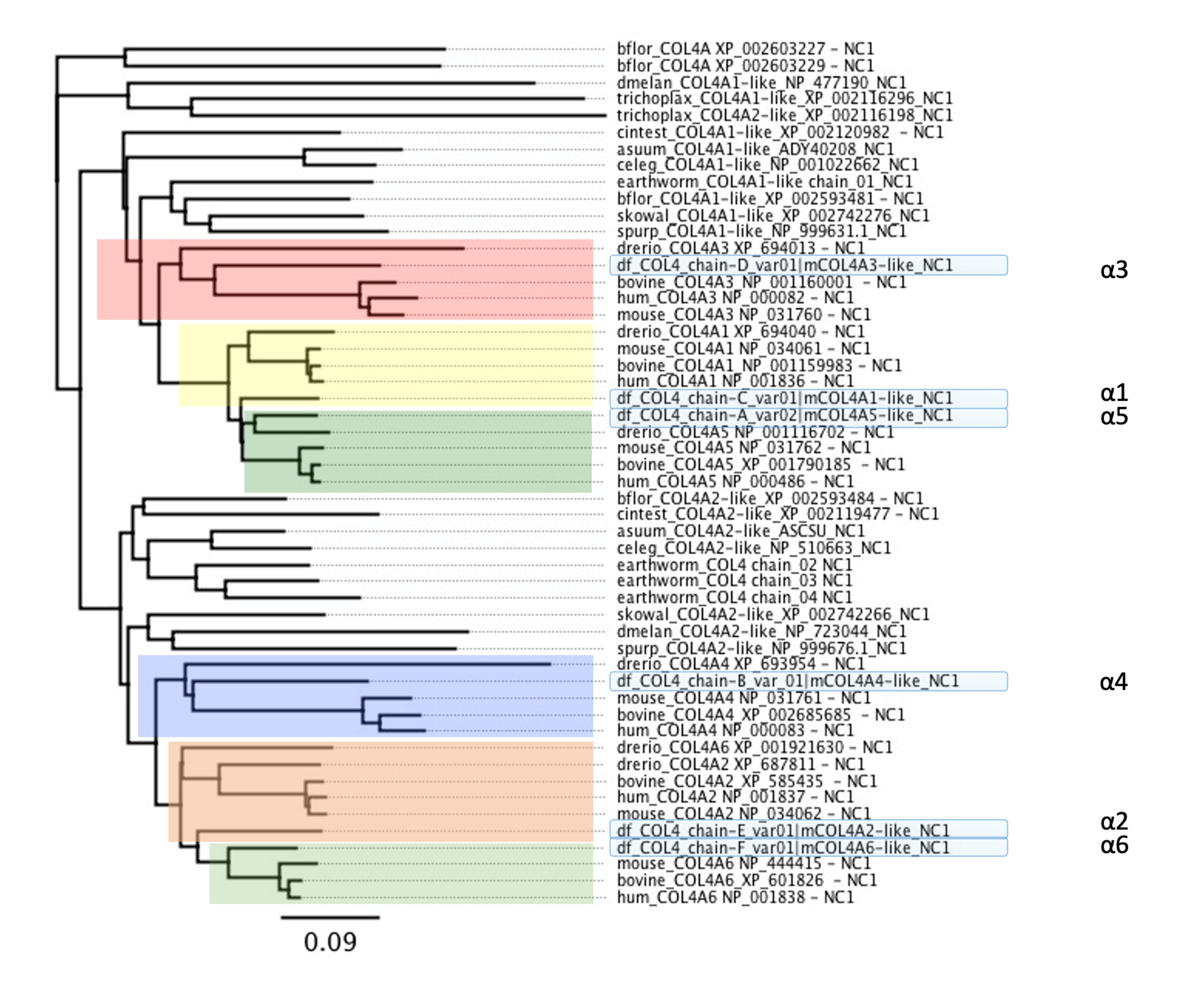
**

**Supporting figure 3. Phylogenetic comparison of Squalus acanthias collagen-IV NC1-domain paralogs to representative metazoan species.**

Full-length sequences of *S. acanthias* (dogfish) were generated via RNASeq and *de novo* transcriptome assembly of dogfish basement membrane isolated from kidney, lens. Transcriptome sequences were confirmed by RT-qPCR. Assignment of dogfish NC1-domains from RNASeq derived full-length chains was made based on known COL4 NC1-domains (a1-a6) from human, mouse, bovine, Drosophila, zebrafish, *C. elegans*, earthworm, *C. intestinalis*, *S. kowalevsky*, *S. purpuratis*. Phylogenetic tree is built using Neighbor-Joining method using Geneious Bioinformatic Software (<https://www.geneious.com/>).

**
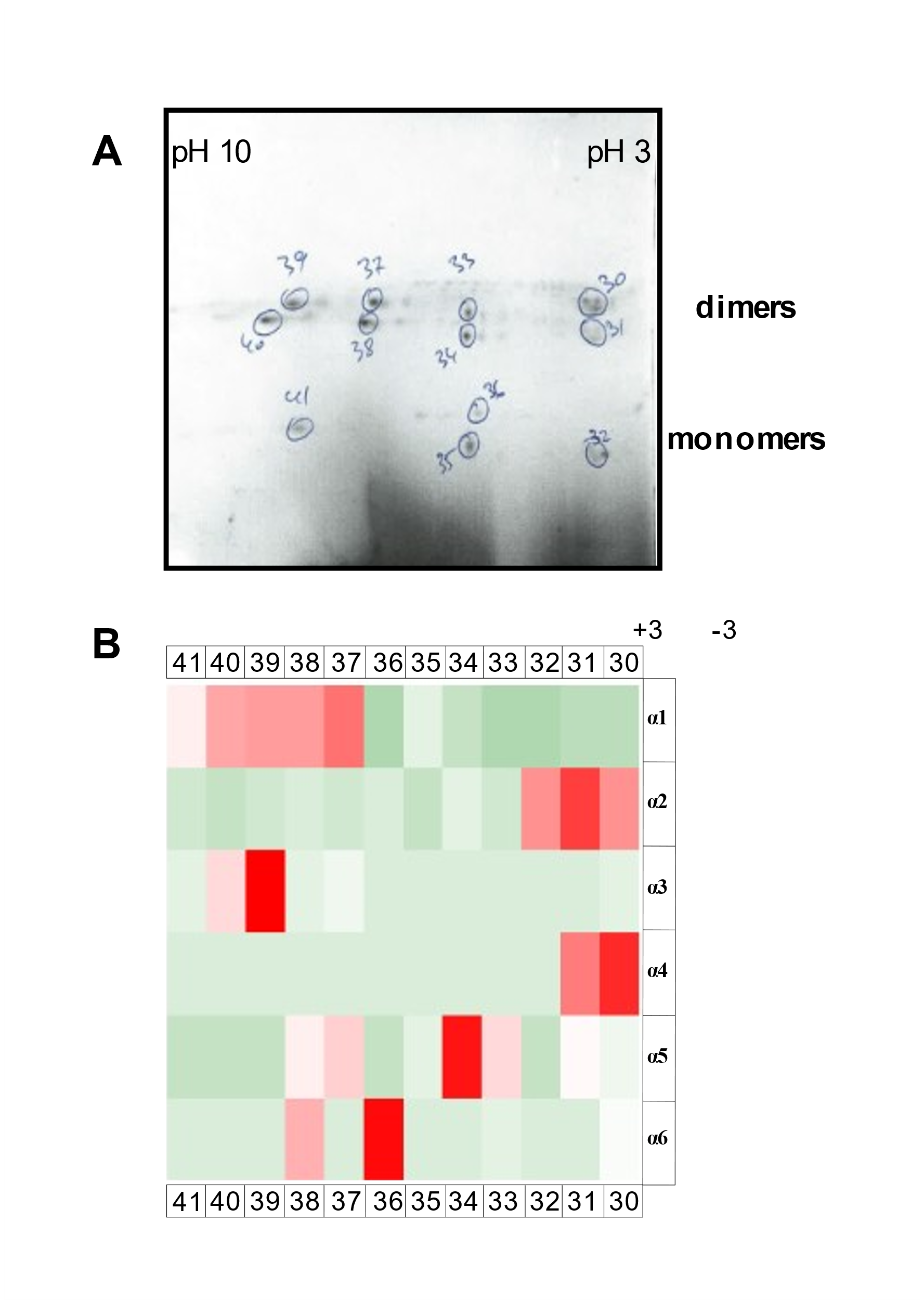
Supporting figure 4.**

**A.** Colloidal Coomassie stained 2D-NEPHGE gel with spots that were excised for mass spectroscopy (indicated with the numbered circles).

**B.** A heat map (heatmapper. ca) built from label free quantification (LFQ) of protein level data. A custom dogfish protein database was used to define chain composition in each 2D spot.


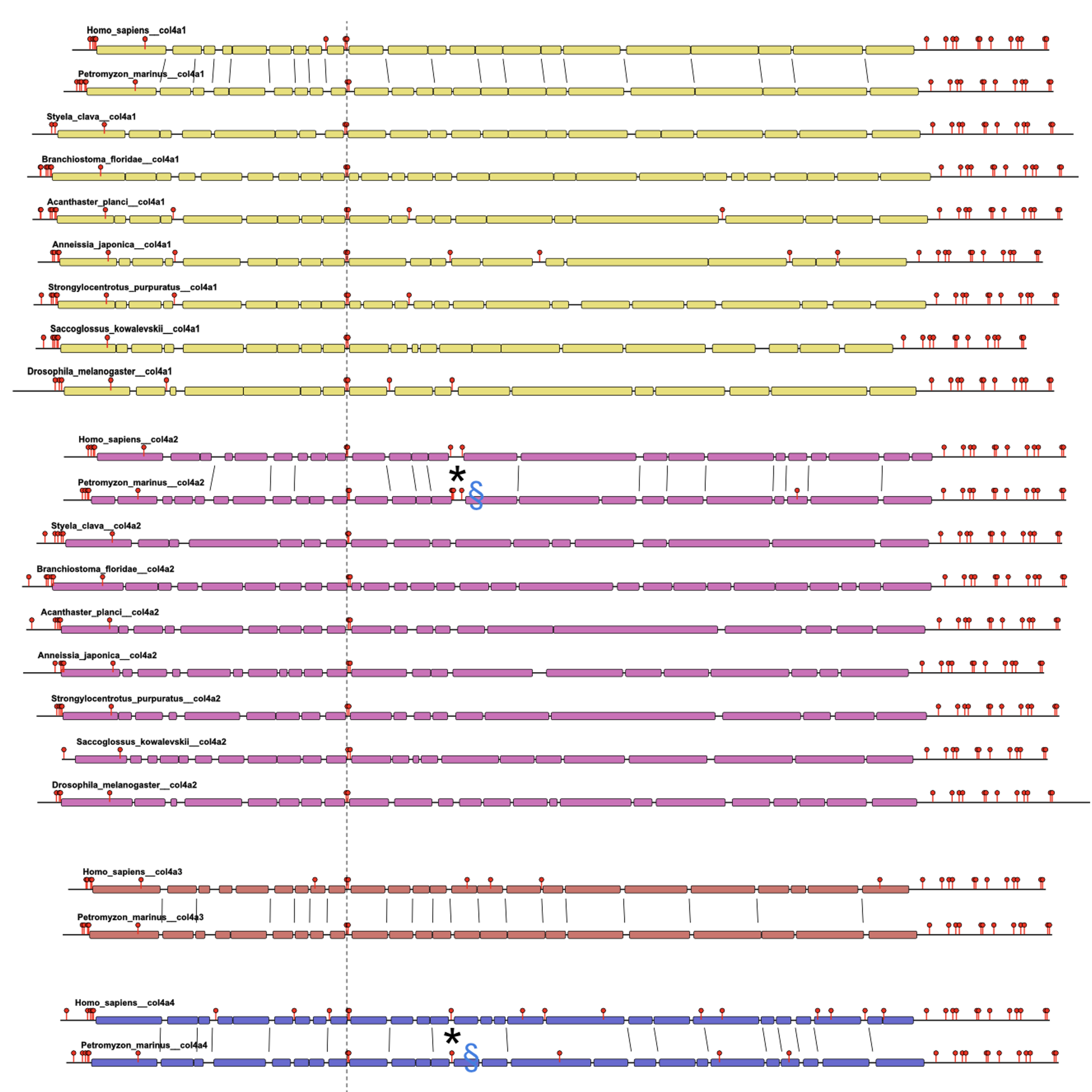


**Supporting Figure 5. Comparison *COL4A*⟨1|2⟩ domain structure within Deuterostomes.**

Predicted collagen domains are shown as blocks with interruptions as lines, the collagens are aligned to the highly conserved cysteine pair. Conservation in placement of the cysteine residues can be seen in the presence of the red flags. The 7S and NC1 domains are found at the N and C termini. Block diagram showing the domain structure of the predicted products of the *COL4A*⟨1|2⟩ loci in basal Deuterostomes. Collagen helical domains are indicated as blocks (Yellow *COL4A*1, pink *COL4A*2). Alignment of the gaps is indicated between the human and cyclostome sequences and the presence of the cys-loop-cys motif is indicated by *. Position of Cys residues and interruptions to the collagen blocks is observed between the human and cyclostome *COL4A*1 and *COL4A*2 genes, consistent with a correct assignment of the genes to their respective *COL4A* gene families.
